# 造血干细胞移植在急性髓系白血病中的应用进展

**DOI:** 10.3760/cma.j.cn121090-20240422-00157

**Published:** 2025-02

**Authors:** 晓璐 朱, 晓军 黄, 昱 王

**Affiliations:** 北京大学人民医院，北京大学血液病研究所，国家血液系统疾病临床医学研究中心，造血干细胞移植治疗血液病北京市重点实验室，北京 100044 Peking University People's Hospital, Peking University Institute of Hematology, National Clinical Research Center for Hematologic Disease, Beijing Key Laboratory of Hematopoietic Stem Cell Transplantation, Beijing 100044, China

## Abstract

急性髓系白血病（AML）是髓系造血干/祖细胞恶性疾病，尽管近年来血液疾病领域新药层出不穷，造血干细胞移植（HSCT）在血液疾病领域中的地位仍不可取代。本文就HSCT在AML的适应证调整和供者选择、预处理方案的更新、移植后复发的新型预防策略、新型靶向药物、免疫治疗联合HSCT改善复发/难治AML患者预后以及老年AML的移植技术优化进行综述。

## Abstract (en)

Acute myeloid leukemia（AML）is a malignant disease of myeloid hematopoietic stem/progenitor cells. Although new drugs have emerged in recent years, the status of hematopoietic stem cell transplantation（HSCT）in AML still remains irreplaceable. This article reviews the adjustment of indications for HSCT in AML, donor selection, and updates of conditioning regimens, new prevention strategies for post-transplant relapse, new targeted drugs, immunotherapy combined with HSCT to improve the prognosis of relapsed and refractory AML, and optimization of transplantation technology for elderly AML.

急性髓系白血病（AML）是髓系造血干/祖细胞恶性疾病，尽管近年来新药层出不穷，造血干细胞移植（HSCT）在血液病治疗中的地位仍不可取代。同时，随着相关技术的不断进步，HSCT在AML中的适应证得到进一步拓展。

一、HSCT在AML的应用现状

近年来，全球异基因造血干细胞移植（allo-HSCT）数量持续增长。2023年美国国际骨髓移植登记中心（CIBMTR）的数据显示，在2021年，AML仍然是接受allo-HSCT的主要病种，自2015年起，每年接受HSCT的AML患者均超过3 000例，远超其他病种且呈逐渐上升趋势。欧洲骨髓移植登记组（EBMT）2022年的统计数据也显示，接受allo-HSCT的AML病例在allo-HSCT中占39％[1]。来自中国骨髓移植登记组（CBMTR）的数据显示，2021年共完成HSCT 18 110例，其中allo-HSCT占70％（12 744例），AML患者占所有HSCT病例的37％（4 964例），是接受HSCT的第一大病种[2]。

造血干细胞供者来源不足一直是白血病治疗领域的世界性医学难题。基于粒细胞集落刺激因子（G-CSF）和抗胸腺细胞球蛋白（ATG）来诱导免疫耐受（北京方案）和基于移植后环磷酰胺（PTCy）诱导免疫耐受（巴尔的摩方案）的单倍体造血干细胞移植（haplo-HSCT）在很大程度上解决了这一关键问题，进一步推动了allo-HSCT的迅猛发展[3]–[8]。CIBMTR的数据显示，从2010年到2018年期间，美国接受haplo-HSCT的例数增长了316％，在所有接受HSCT病例中占比接近1/4。EBMT的资料显示，2022年，689个欧洲中心报告了46 143次HSCT，包括19 011次（41.2％）allo-HSCT和27 132次（58.8％）auto-HSCT，其中近4 000次为haplo-HSCT，数量呈上升趋势[1]。CBMTR的数据显示，2021年在allo-HSCT中，haplo-HSCT占63％（7 977例）；2020至2021年共8 853例AML患者接受HSCT，其中5 511例（62％）为haplo-HSCT，亲缘全相合供者HSCT（MSD-HSCT）1 721例（19％）[2]。

二、AML的HSCT适应证和供者选择

在上述三个大型登记组资料中，AML占allo-HSCT适应证的首位。随着对AML的危险分层不断细化，预后良好组AML的移植适应证得到进一步明确；预后中危组和预后高危组的供者选择有了进一步拓展。

（一）预后良好组AML的移植适应证

对于AML（不包括急性早幼粒细胞白血病），根据最新美国国立综合癌症网络（NCCN）[9]和欧洲白血病网络（ELN）[10]推荐，预后良好组一般无须在第一次完全缓解（CR1）期进行allo-HSCT。随着对AML的危险分层不断细化，基于化疗早期微小残留病（MRD）动态变化筛选出高危患者，这些患者在CR1期接受allo-HSCT可获益。

《中国异基因造血干细胞移植治疗血液系统疾病专家共识（2021版）》[11]推荐的allo-HSCT适应证包括：①巩固2个疗程后RUNX1∷RUNX1T1转录本水平较基线下降<3个log或在6个月内失去主要分子学缓解（MMR）；②伴有CBFβ∷MYH11的AML，巩固2个疗程后任意时间CBFβ∷MYH11/ABL>0.1％；③伴有CBFβ∷MYH11的AML，合并伴有D816 KIT突变；④伴有CEBPA双突变的AML，巩固2个疗程后任意时间流式细胞术MRD阳性；⑤伴有NPM1突变AML患者化疗后MRD持续阳性。

（二）预后中危组AML的移植适应证

对于预后中危组AML，根据MRD动态危险度分层调整移植适应证，可改善患者预后。NCCN推荐具有中等或不良细胞遗传学风险的患者可能获益于CR1期HSCT[9]。《成人急性髓系白血病（非急性早幼粒细胞白血病）中国诊疗指南（2023年版）》[12]进一步细化，对于MRD持续阳性或MRD由阴性转为阳性（尤其是巩固治疗完成后MRD阳性）的患者，建议进行allo-HSCT。另外，预后中危组1个疗程化疗后流式细胞术MRD阳性患者，建议行allo-HSCT。

（三）预后中危组和预后高危组AML的供者选择

目前，国内外指南均推荐allo-HSCT作为预后高危组AML患者缓解后的首选治疗，而且在CR1期即进行allo-HSCT。来自Southwest Oncology Group[13]、EORTC-LG/GIMEMA[14]、EAST German Study Group[15]和German Hemoblastosis Group[16]等研究组的随机对照临床研究结果显示，高危AML患者在CR1期接受HLA相合同胞供者（MSD）或无关供者allo-HSCT，其疗效显著优于化疗和auto-HSCT。相比于高危AML，allo-HSCT在中危AML CR1期的地位随着移植技术的发展而逐步得到确认，越来越多的数据表明中危AML CR1患者接受allo-HSCT后无白血病生存（LFS）期和总生存（OS）率可获得显著提高[16]–[17]。

allo-HSCT已被证实能够改善预后中危组和预后高危组的AML患者的预后，但供者类型对移植预后的影响尚不明确。近年来随着haplo-HSCT技术的不断成熟和完善，haplo-HSCT的疗效得到明显改善。本中心报告的“北京方案”haplo-HSCT治疗756例恶性血液病10年长期随访结果显示，100％的患者获得完全供者植入，重度急/慢性移植物抗宿主病（GVHD）累积发生率低于15％，3年非复发死亡率（NRM）为18％，标危、高危患者3年无病生存（DFS）率分别为68％、49％[18]。该结果证实，预后中高危组AML CR1患者在缺乏MSD时，“北京方案”haplo-HSCT可作为替代选择。

研究者从小鼠模型中发现，haplo-HSCT可通过促进细胞凋亡减少和细胞毒性细胞因子分泌（包括T细胞或自然杀伤细胞分泌的肿瘤坏死因子-α、干扰素-γ、成孔蛋白和CD107a），从而发挥更强的移植物抗白血病（GVL）效应[19]。体外研究结果表明haplo-HSCT组的细胞毒性T淋巴细胞比例比同期MSD组更高[19]。临床研究结果提示，haplo-HSCT在成人及儿童高危AML、复发/难治AML（R/R AML）患者中均有更强的GVL效应[20]–[22]。

对预后中危组AML CR1患者，Lv等[23]进行的前瞻临床试验结果显示，haplo-HSCT组移植后3年DFS率、OS率（74.3％对47.3％，*P*＝0.0004；80.8％对53.5％，*P*＝0.0001）均优于化疗组，是降低白血病复发、提高生存的独立因素。对于预后中危组和高危组的AML CR1患者，haplo-HSCT组与MDS组具有相似的移植后3年DFS率（74％对78％，*P*＝0.34）、OS率（79％对82％，*P*＝0.36）、NRM（13％对8％，*P*＝0.13）以及累积复发率（CIR，15％对15％，*P*＝0.98）[24]。Yu等[21]报告的多中心队列研究结果中，预后高危组AML患者CR1期接受haplo-HSCT的移植后MRD阳性率显著低于MSD-HSCT组（18％对42％，*P*<0.001），两组移植后3年CIR（14％对24％，*P*＝0.101）、OS率（72％对68％，*P*＝0.687）、DFS率（71％对66％，*P*＝0.579）相似。另有研究显示haplo-HSCT可克服FLT3-ITD、TP53的不良预后[25]–[26]。

本中心报告的haplo-HSCT治疗1 199例CR1期急性白血病患者的前瞻性多中心临床研究[27]结果显示：供患者年龄偏大、女供男组合、供患者ABO血型不合为移植相关死亡的3个不良预后因素，与是否MSD无关；以供患者年龄、性别、血型相合为核心的积分体系而非经典的HLA决定移植预后，即危险积分高的MSD-HSCT疗效差于危险积分低的haolo-HSCT，挑战了MSD作为首选供者的经典法则。更重要的是，确认了在老年白血病中年轻供者haplo-HSCT优于年老供者MSD-HSCT。随着≥50岁老年急性白血病患者的增多，越来越多的老年白血病患者需要接受allo-HSCT，然而匹配的同胞供者年龄偏大。北京大学血液病研究所发起的多中心研究显示，年轻haplo-HSCT组、年老MSD-HSCT组移植后3年移植相关死亡率（TRM）分别为9％、26％（*P*＝0.023），CIR分别为6％、17％（*P*＝0.066），OS率分别为85％、58％（*P*＝0.003），LFS率分别为85％、56％（*P*＝0.001），Ⅱ/Ⅳ度急性GVHD（aGVHD）的累积发生率分别为26％、35％（*P*＝0.23），慢性GVHD累积发生率分别为37％、24％（*P*＝0.19）[28]。一项纳入24项研究共11 359例患者的Meta分析结果显示，haplo-HSCT治疗恶性血液病可取得与MSD-HSCT相当的移植后1年CIR（*P*＝0.180）和NRM（*P*＝0.910）[29]。

基于以上研究结果，对于中高危AML患者在CR1期即可进行allo-HSCT，首选MSD-HSCT，在缺乏MSD时，可选haplo-HSCT或其他可行移植模式。对于老年中高危AML CR1患者，有经验的移植中心可优先选择haplo-HSCT[11]。

三、allo-HSCT预处理方案

来自CBMTR数据显示，2020至2021年期间，在allo-HSCT预处理方案中经典的Bu/Cy（白消安+环磷酰胺）方案占57％[2]；恶性血液病移植预处理方案中，Bu/Cy方案亦占首位（占62％）[2]。我国的多中心随机对照Ⅲ期临床研究结果表明，在haplo-HSCT中，Bu/Flu（白消安+氟达拉滨）方案比Bu/Cy方案具有更低的1年TRM和3级预处理毒性（RRT）发生率（7.2％对14.1％，*P*＝0.041；0％对4.7％，*P*＝0.002），5年CIR相似（17.9％对14.2％，*P*＝0.670）[30]。来自意大利的多中心随机对照Ⅲ期临床研究也证实Bu/Flu方案的TRM低于Bu/Cy方案[31]。

对于老年患者（年龄≥55岁）行haplo-HSCT，本中心进行的一项前瞻性Ⅱ期临床试验[32]结果表明，在Bu/Flu/Cy/ATG（白消安+氟达拉滨+环磷酰胺+抗胸腺细胞球蛋白）预处理方案下，所有患者均获得粒细胞植入（完全供者嵌合），移植后100 d内Ⅱ～Ⅳ度aGVHD发生率为22.0％，移植后1年OS率、LFS率分别为63.5％、60.2％，TRM为23.3％。使用倾向评分匹配方法，上述结果与Bu/Cy/ATG（白消安+环磷酰胺+抗胸腺细胞球蛋白）预处理方案相当，提示Bu/Flu/Cy/ATG预处理方案适用于老年患者haplo-HSCT。

对于首次haplo-HSCT后发生植入失败的患者，黄晓军团队探索出一种包括减低剂量预处理、更换供者、良好支持治疗的全新方案。二次移植预处理方案：−6 d ～ 2 d给予Flu 30 mg·m^−2^·d^−1^静脉注射，−5 d和−4 d给予Cy 1 000 mg·m^−2^·d^−1^静脉注射。在二次移植前进行供者特异性抗体（DSA）检测，优先考虑DSA阴性供者。在既往研究报告中，GVHD的预防包括巴利昔单抗（20 mg、−1 d和+4 d）、环孢素A（血浓度150～250 µg/L）和霉酚酸酯。由于aGVHD发生率较高，因此在+15 d追加1剂巴利昔单抗（共3剂），以加强对GVHD的预防。30例患者中，29例患者获得粒细胞植入，中性植入中位时间为11（8～24）d，移植后1年OS率、LFS率分别为60％、53.3％，CIR、TRM分别为6.7％、33.3％。上述数据显示了该方案在植入和生存方面的优势，可作为恶性血液病患者首次allo-HSCT植入失败的一种挽救治疗方案[33]。

四、移植后复发的新型预防策略

白血病复发仍是allo-HSCT失败的主要原因之一。CIBMTR的资料显示，复发在非同胞相合移植后的100 d内、100 d后的死亡原因中分别占29％、57％，在无关供者移植后100 d内、100 d后的死亡原因中分别占23％、46％。有效防治移植后复发是提高移植疗效的关键。减停免疫抑制剂、化放疗和二次移植作为传统的复发防治手段仍然适用，但疗效有限。供者淋巴细胞输注（DLI）、分子靶向药物、细胞因子治疗等新方法近年来得到快速发展[34]–[36]。

（一）DLI

随着DLI技术的不断改进，逐步形成了危险度分层体系指导下的基于haplo-HSCT后新型DLI的白血病复发防治体系。对于MRD阳性患者进行干预性DLI，对于移植前处于难治或复发状态的患者进行预防性改良DLI[37]。与单次化疗联合DLI患者比较，基于DLI后MRD和GVHD双指标指导多次化疗联合DLI患者的再次复发率较低（22％对56％，*P*<0.001），LFS率、OS率较高（71％对35％，*P*<0.001；78％对44％，*P*<0.001）[38]。该预防体系对于移植前CR或R/R AML，均可降低移植后复发、改善生存。

（二）分子靶向药物

分子靶向药物对移植后复发的预防作用也得到验证。国内一项开放标签、多中心、随机、Ⅲ期试验的5年长期随访研究[39]表明：索拉非尼组的1年CIR为7.0％（95％ *CI* 3.1％～13.1％），对照组为24.5％（95％ *CI* 16.6％～33.2％；*HR*＝0.25，95％ *CI* 0.11～0.57，*P*＝0.001）。IDH1抑制剂艾伏尼布和IDH2抑制剂恩西地平已经应用于R/R AML中[40]–[41]，其在移植后复发患者的临床试验正在进行（NCT03728335、NCT03564821）。一项来自德国的回顾性研究纳入32例移植后复发髓系肿瘤患者，应用BCL2抑制剂维奈克拉与阿扎胞苷（13例）或地西他滨（DAC）（19例）联用，平均随访8.4个月，客观缓解率（ORR）为47％，25例（78％）死亡，7例存活，预期中位OS期为3.7个月[42]。维奈克拉为基础的其他联合治疗（如克拉屈滨）在移植后复发患者中的疗效亦得到证实[43]–[44]。

（三）去甲基化药物

多种去甲基化药物在移植后维持治疗中得到应用。去甲基化药物阿扎胞苷被证实可上调由表观遗传沉默相关肿瘤基因表达，诱导CD8^+^ T细胞反应，从而增强GVL效应，而不增加GVHD风险[45]–[46]。数项研究提示低剂量阿扎胞苷作为R/R AML/骨髓增生异常综合征（MDS）患者移植后维持治疗安全有效[47]–[51]。低剂量DAC联合维奈克拉作为移植后维持治疗同样安全有效[52]。DAC联合DLI亦可作为高危AML移植后复发的预防策略[53]。多中心随机研究表明，FLT3抑制剂（索拉菲尼或吉瑞替尼）或低剂量DAC联合G-CSF可显著提高FLT3阳性或高危AML患者的生存率[39],[50],[54]–[56]。

（四）细胞因子

预防性α干扰素治疗通过免疫激活被提示是消除移植后MRD的有效方法，其良好的耐受性和便捷性为其在移植后患者的应用提供了可行性[57]。我中心基于两项临床注册研究（NCT02185261、NCT02027064）评估α干扰素抢先治疗在接受allo-HSCT的AML患者中的长期疗效，接受α干扰素抢先治疗组6年CIR、NRM、DFS率和OS率分别为13.0％、3.9％、83.1％和88.3％，慢性移植物抗宿主病（cGVHD）、严重cGVHD的6年累积发生率分别为66.2％、10.4％，初步提示α干扰素抢先治疗是安全、有效的预防疾病复发手段[58]。

五、展望

（一）新型靶向药物、免疫治疗联合HSCT提高R/R AML预后

尽管近年来AML的治疗手段不断发展，但R/R AML的预后极差，5年OS率仅为10％[59]。目前主要的治疗选择包括选择无交叉耐药的新药组成联合化疗方案、耐药逆转剂、靶向药物、免疫治疗等，而这些手段的目的是获得更深的疾病缓解、更大程度降低肿瘤负荷，后续桥接allo-HSCT仍是提高R/R-AML治愈率的重要策略，allo-HSCT仍是唯一可能治愈的手段[9]–[10]。

多种靶向药物的问世，让R/R AML患者有机会桥接HSCT，为他们带来新的曙光。ivosidenib、gilteritinib、flotetuzumab等已被发现可成功用于R/R AML患者单独治疗或与allo-HSCT联合治疗（尤其是haplo-HSCT）。一项多中心研究[60]纳入17例合并IDH1突变的R/R AML患者，11例（65％）获得CR、5例（29％）获得完全缓解伴血液学不完全恢复（CRi）、1例获得形态学无白血病状态（MLFS）。移植后6、12个月的OS率分别为76.5％、47.1％。该结果表明ivosidenib单药可使合并IDH1突变的AML获得缓解，使这些患者有机会桥接HSCT。Zucenka等[61]应用gilteritinib与维奈克拉及低剂量阿糖胞苷和放线菌素D联合方案治疗20例合并FLT3突变的R/R AML患者，ORR达到95％（19/20），完全缓解率和完全缓解伴血小板不完全恢复（CR + CRp）率达75％（15/20），11例患者桥接allo-HSCT。一项多中心研究[62]报道了flotetuzumab（靶向CD3和CD123的双特异性抗体）在R/R AML中的应用，88例患者中10例（11.7％）获得CR或部分血液学恢复的CR（CRh），ORR为13.6％（12/88）；在原发性诱导失败（PIF）或初始缓解期<6个月（早期复发）的患者中，CR+CRh率为26.7％，ORR为30.0％，6、12个月OS率分别为42％、20％。

中国学者开展的多项新型细胞免疫治疗取得初步良好结果。C型凝集素样分子1（CLL1）嵌合抗原受体T细胞（CAR-T）治疗成人R/R AML的临床研究初步取得了令人鼓舞的疗效和良好的安全性[63]。10例患者中7例获得CR或CRi。相似的疗效在儿童R/R AML中也得到初步证实。CAR-T治疗后1个月内，8例患者中4例获得MLFS和MRD阴性，1例为MLFS合并MRD阳性，1例达到CRi但MRD阳性，1例部分缓解（PR），1例保持疾病稳定（SD）状态但CLL1阳性AML幼稚细胞清除。8例患者中有6例桥接allo-HSCT[64]。CD38-CART在6例allo-HSCT后复发的AML患者中的安全性及疗效也初步得到验证：输注后4周评效，4例（66.7％）患者达到CR或CRi[65]。靶向CD33的CAR-NK细胞疗法在R/R AML患者中的Ⅰ期临床试验也取得可喜的初步结果：在第28天评估时，10例患者中6例获得MRD阴性CR[66]。

综上，上述新型治疗手段拓展了R/R AML的移植适应证。展望未来，HSCT和上述新技术的结合有望使R/R AML整体疗效更上一层楼[67]。

（二）老年AML的移植技术优化

AML主要发病人群为中老年人，中位发病年龄为67岁[68]。老年AML患者具有脏器功能衰退、骨髓储备功能差、免疫功能衰退、个体化差异较大等特点，治疗耐受性差，总体缓解率低，治疗相关死亡率高，生存时间短。CIBMTR数据表明，2018至2021年，每年有800例以上大于65岁老年患者接受allo-HSCT，在所有allo-HSCT中占比近1/3并呈上升趋势。CBMTR数据表明大于65岁老年患者接受allo-HSCT的比例已由2008至2016年的0.4％上升至2017至2019年的4.0％。

近年来，随着移植预处理方案的优化、GVHD预防策略的完善、支持治疗的改进以及评估手段的增强，越来越多的老年患者接受allo-HSCT并获益。北京大学血液病研究所牵头的国内多中心研究证实了年轻HID较年老MSD在老年患者的优势和安全性[28]。在预处理方案的选择上，Bu/Flu/Cy/ATG作为老年患者haplo-HSCT预处理方案是可行的[32]。2017年，北京大学血液病研究所已经可以常规为65岁以下患者行allo-HSCT。如今，allo-HSCT患者的年龄上限提高至77岁。随着老年AML对HSCT的需求与日俱增，如何提高老年患者HSCT的安全性和生活质量仍是目前所面临的一个巨大挑战。

六、结语

allo-HSCT的快速发展正逐渐革新AML治疗模式，移植获益人群不断扩大。haplo-HSCT在中高危AML及老年AML中的地位逐步攀升。靶向治疗、免疫治疗与HSCT的联合有望进一步改善血液系统疾病患者的长期生存和生活质量。
